# Cortex Mori Radicis Mitigates Inflammation and Fibrosis in Pulmonary Fibrosis Through PI3K/AKT Pathway Suppression

**DOI:** 10.1155/carj/8459298

**Published:** 2026-06-28

**Authors:** Tianxiang Qi, Bingfeng Ma, Yuanyuan Peng, Yafeng Liu, Jianqiang Guo, Xingyu Yang, Xuansheng Ding, Dingfei Ren, Dong Hu

**Affiliations:** ^1^ The First Affiliated Hospital of Anhui University of Science and Technology (Huainan First People’s Hospital), School of Medicine, Anhui University of Science and Technology, Huainan, China, aust.edu.cn; ^2^ Anhui Occupational Health and Safety Engineering Laboratory, School of Medicine, Anhui University of Science and Technology, Huainan, China, aust.edu.cn; ^3^ School of Pharmacy, China Pharmaceutical University, Nanjing, China, cpu.edu.cn; ^4^ Occupational Control Hospital of Huaihe Energy Group, Huainan, China; ^5^ Key Laboratory of Industrial Dust Deep Reduction and Occupational Health and Safety of Anhui Higher Education Institutes, School of Medicine, Anhui University of Science and Technology, Huainan, China, aust.edu.cn

**Keywords:** Cortex Mori Radicis, herbal medicine, molecular docking, network pharmacology, pulmonary fibrosis

## Abstract

Early in the pathogenesis of pulmonary fibrosis (PF), there are multiple inflammatory cell infiltrations in the damaged lung tissue. When lung injury persists, inflammatory cytokines prompt local fibroblasts migration and hyperproliferation, triggering abnormal deposition of extracellular matrix in the lung interstitium. This excessive repair leads to interstitial cell reorganization, triggering lung tissue fibrosis and further activation of inflammatory cells. Therefore, modulation of inflammatory mediators is of great significance in the treatment and prevention in the process of fibrosis. Cortex Mori Radicis (CMR) is a traditional Chinese herb with anti‐inflammatory and antifibrotic properties. In this study, we investigated the therapeutic effects of CMR on bleomycin‐induced PF using in vivo and in vitro models. In vivo experiments showed that CMR treatment significantly reduced inflammation, attenuated fibrosis, and alleviated lung function decline. In vitro, CMR inhibited migration, proliferation, and epithelial–mesenchymal transition (EMT) in A549 lung epithelial cells. Network pharmacological analysis identified 25 bioactive components and 10 key therapeutic targets in CMR, with the PI3K/AKT signaling pathway emerging as the core regulatory mechanism. Subsequent in vivo validation confirmed that CMR could inhibit the activation of the PI3K/AKT pathway. In conclusion, CMR exerts protective effects against PF by modulating the PI3K/AKT pathway, thereby attenuating inflammation and fibrotic remodeling. This study provides both pharmacodynamic evidence and mechanistic insight supporting the clinical potential of CMR and underscores the advantages of multitargeted intervention strategies offered by traditional Chinese medicine in the treatment of PF.

**Highlights**
• Cortex Mori Radicis (CMR) was first found to treat pulmonary fibrosis (PF).
• CMR reduces lung inflammation and PF by inhibiting the PI3K/AKT signaling pathway.
• The key active ingredients and targets of CMR for the treatment of PF were screened by network pharmacology.

## 1. Introduction

Idiopathic pulmonary fibrosis (IPF) is a fatal, chronic, progressive interstitial lung disease of unknown etiology, characterized by excessive extracellular matrix (ECM) deposition, alveolar epithelial cell injury, and fibroblast activation [1, 2]. Its pathogenesis involves dysregulated wound healing driven by complex interactions between profibrotic mediators such as TGF‐β1 [3] and aberrant activation of signaling pathways like PI3K/AKT [4], which promote fibroblast proliferation and resistance to apoptosis [5]. Current therapeutic strategies, primarily limited to antifibrotic agents like pirfenidone and nintedanib, demonstrate modest efficacy in slowing disease progression but fail to halt or reverse fibrotic remodeling [6]. Given these limitations, traditional Chinese medicine (TCM) has garnered attention as a complementary approach for managing IPF. TCM emphasizes multitarget therapies that may reduce inflammation, improve lung function, and enhance quality of life through personalized herbal formulations, while potentially offering fewer systemic side effects compared to conventional treatments [7].

Cortex Mori Radicis (CMR), a traditional herbal medicine derived from the root bark of Morus alba L., is known for its sweet and cold properties and its affinity for the lung meridian [8, 9]. It exhibits multiple pharmacological activities, including anti‐inflammatory, antioxidant, and immunomodulatory effects [10, 11]. Previous studies have shown that CMR alleviates airway inflammation in asthma models by inhibiting Th2 cytokines [12] and that its phenolic components improve epithelial–mesenchymal transition (EMT) in renal fibrosis [13]. Given its multifaceted pharmacological profile, including anti‐inflammatory and immunomodulatory properties, coupled with emerging evidence of its efficacy in mitigating fibrosis‐related pathways, CMR presents a promising candidate for further exploration of its therapeutic potential in addressing IPF.

This study aims to investigate the anti‐inflammatory and antifibrosis effects of CMR on bleomycin (BLM)‐ and TGF‐β1‐induced pulmonary fibrosis (PF) in in vivo and in vitro models. Focusing on the PI3K/AKT signaling pathway, this research elucidates the molecular mechanisms by which CMR mitigates PF through anti‐inflammatory effects, EMT inhibition, and collagen deposition reduction. Additionally, we integrate network predictions to uncover the bioactive components and molecular targets of CMR in PF. Our findings provided the potential mechanism of CMR and multitarget antifibrotic candidate treatment.

We utilized a BLM‐induced PF model, which is widely accepted for preclinical evaluations due to its ability to mimic key fibrotic features including inflammation, EMT, and ECM deposition. It is important to note that while this model provides valuable insights, its progression differs from the spontaneous, age‐related pathogenesis of IPF. This limitation is acknowledged, and our findings should be interpreted within the context of this experimental approximation.

## 2. Materials and Methods

### 2.1. Materials and Reagents

BLM was obtained from MCE (#HY‐17565A). Primary antibodies included β‐actin (ABclonal, #AC026), GAPDH (ABclonal, #AC001), N‐cadherin (CST, #13116T), E‐cadherin (Proteintech, #20874‐1‐AP), Vimentin (Servicebio, #GB11192‐100), Collagen I (Servicebio, #GB114197‐100), Collagen III (Servicebio, #GB111629‐100), α‐SMA (CST, #19245T), PI3K (ABclonal, #A4992), AKT (CST, #9271), p‐PI3K (ABclonal, #AP0427) and p‐AKT (Cell Signaling, #4060T).

### 2.2. Preparation of CMR

CMR was purchased from Xi’an Lisen Biotechnology Co., Ltd (Xi’an, China, #LS202401210). The specific preparation process is as follows: the dried mulberry bark roots are crushed, 95% ethanol is added, heated, and then refluxed to extract three times, 60 each time min, gauze filtration, heating, decompression, concentration at 50°C–60°C to recover ethanol, freeze‐drying the resulting extract, storing at −20°C, removing when needed, precision weighing, and diluted with distilled water to the desired concentration.

### 2.3. Animal Experiments

Male C57BL/6J mice (6–8 weeks old, 20–24 g) were obtained from SKBEX Biotechnology (HeNan, China) and housed at the Experimental Animal Center of the School of Medical Sciences, Anhui University of Science and Technology. The mice were maintained in a controlled environment at 20 ± 2°C and 55 ± 5% humidity with a 12‐h light/dark cycle, provided with continuous access to water, and fed a standard diet compliant with the Chinese National Laboratory Mouse Feeding Standards. After a 3‐day acclimatization period, the mice were weighed daily and randomly assigned to three groups (*n* = 5 per group): Normal Saline Control (NC), BLM, and Bleomycin + Cortex Mori Radicis (BLM + CMR). Mice in the NC group were orally gavaged with an equal volume of normal saline, while those in the BLM group received an intratracheal injection of BLM (5 mg/kg) and subsequently orally gavaged with normal saline from the second day until Day 28. The BLM + CMR group received an intratracheal injection of BLM (5 mg/kg) and, starting from the second day, were treated with CMR via oral gavage (200 mg/kg, once daily) for 28 days. On the day following the final treatment, mice were euthanized by cervical dislocation after inhalation anesthesia with 3% isoflurane (RWD, #R510‐22‐10). All experiments adhered to the ARRIVE guidelines and were approved by the Biomedical Ethics Committee of Anhui University of Science and Technology (no. HX‐001). The CMR dose (200 mg/kg, once daily) was selected based on previous preclinical studies. Kim et al. demonstrated that 50–200 mg/kg of CMR extract effectively attenuated airway inflammation in a mouse asthma model without observable toxicity [12].

### 2.4. Cell Culture and In Vitro Fibrosis Model

The A549 human lung epithelial cell line was purchased from the Institute of Biochemistry and Cell Biology, Chinese Academy of Sciences (Shanghai). Cells were cultured in RPMI‐DMEM medium (Gibco) supplemented with 10% fetal bovine serum (FBS, Gibco), 100 U/mL penicillin, and 100 U/mL streptomycin (Beyotime, #C0222). The cells were maintained in a humidified incubator at 37°C with 5% CO_2_. An in vitro EMT and fibrosis model was established by treating A549 cells with 5 ng/mL TGF‐β1 for 24 h.

### 2.5. Lung Tissue Histopathological Staining and Analysis

In accordance with a previously described protocol [14], lung tissues were sectioned into slices of 5–7 μm thickness. These sections were stained with hematoxylin and eosin (H&E) and Masson’s trichrome to visualize inflammation and fibrosis under a microscope. Inflammation in lung tissues was scored using the Szapiel method [15], and the sections were analyzed using ImageJ software. This analysis included the Ashcraft scoring system (graded from 0 to 8) [16] and the quantification of collagen‐positive areas.

### 2.6. Mouse Lung Function Assessment

Lung function in mice was assessed using whole‐body plethysmography (WBP). Mice were placed in the experimental chamber, allowed to acclimate for 30 min with a diversion flow, and once the respiratory signal stabilized, respiratory data were recorded for 5–10 min. Respiratory parameters measured included inspiratory time (Ti, seconds), expiratory time (Te, seconds), peak inspiratory flow (PIF, mL/s), peak expiratory flow (PEF, mL/s), respiratory frequency (F, breaths per minute), tidal volume (Vt, mL), minute ventilation (MV, mL), alveolar ventilation (AV, L), expiratory flow at 50% of vital capacity (EF50, mL/s), end‐inspiratory pause (EIP), end‐expiratory pause (EEP), relaxation time (TR), and enhanced pause (PenH). For this study, Ti, Te, PIF, F, Vt, and EF50 were selected as indicators of lung function [17, 18].

### 2.7. RNA Extraction and Real‐Time Quantitative PCR

Total RNA was extracted from collected mouse lung tissues and cells according to a previously described protocol [14]. RNA concentration and purity were assessed using a spectrophotometer, with RNA considered pure if the absorbance ratio at 260 nm/280 nm was between 1.8 and 2.0. The RNA was reverse‐transcribed into cDNA using the RevertAid First Strand cDNA Synthesis Kit (Thermo Fisher Scientific, #K1622). RT‐qPCR was performed using the 2X Universal SYBR Green Fast QPCR Mix (ABclonal, #RK21204). Relative gene expression was calculated using the 2^−ΔΔCt^ method, with GAPDH as the endogenous reference. Primers used in this study are listed in Supporting Table 1.

### 2.8. Protein Extraction and Western Blot Analysis

Proteins were extracted from collected lung tissues and cells according to a previously described protocol [14]. Subsequently, 50 μg of protein samples was subjected to heating for denaturation and mixed with loading buffer. The proteins were separated by SDS‐PAGE and transferred onto a 0.2‐μm PVDF membrane (Immobilon, #ISEQ00010). The membrane was blocked with 5% skim milk at room temperature to prevent nonspecific binding, followed by washing with TBST. The membrane was then incubated with specific primary antibodies overnight at 4°C, followed by TBST washes. Secondary antibodies were applied for 1 h at room temperature. Afterward, the membrane was incubated with ECL substrate (Millipore, #WBKLS0100) for 30 s and visualized using a chemiluminescent imaging system (Amersham ImageQuant 800, #29399481). Protein bands were analyzed using ImageJ software (V1.8.0) to quantify the intensity of the bands.

### 2.9. Cell Viability Assay

A549 cells were seeded at a density of 3000 cells per well in a 96‐well plate and treated with various concentrations of CMR (7.8–1000 μg/mL) for 24 and 48 h, according to the manufacturer’s instructions for the CCK‐8 assay kit. The effect of CMR on cell viability was assessed. Absorbance was measured at 450 nm using a microplate reader. Cell viability was calculated using the formula: cell viability = (OD control − OD blank)/(OD sample − OD blank).

### 2.10. Wound Healing Assay

A549 cells in the logarithmic growth phase were seeded into 6‐well plates for wound healing assays. Cells were cultured in DMEM medium supplemented with 10% FBS containing CMR at concentrations of 250 and 500 μg/mL and/or 5 ng/mL TGF‐β1 until they reached 80% confluence. Three linear scratches were made along the bottom of the culture dish using a sterile 10‐μL pipette tip. Cells were washed with PBS to remove debris, followed by the addition of fresh serum‐free DMEM medium. Images were captured at 0, 12, and 24 h using an inverted microscope (Leica 3000). Cell migration distance was measured using ImageJ software (Version 1.8.0) to calculate the migration rate. The scratch healing rate (%) was calculated using the formula: scratch healing rate (%) = scratch area at 0 h (scratch area at 0 h−scratch area at 24 or 48 h) × 100%.

### 2.11. Colony Formation Assay

Cells were seeded at a density of 1000–1500 cells per well in small culture dishes and cultured in DMEM medium supplemented with different concentrations of CMR (250 and 500 μg/mL) and/or TGF‐β1 (5 ng/mL) under standard conditions (37°C, 5% CO_2_). The medium was replaced every 3 days to maintain optimal growth conditions. Cells were monitored for approximately 10 days, with the experiment concluding when most colonies contained over 50 cells. Cells were then fixed and stained with a mixture of 4% paraformaldehyde and 0.01% crystal violet. Colonies were visualized and documented using an inverted microscope.

### 2.12. Collection of CMR Active Components and Corresponding Targets

Active components of CMR were identified using the TCMSP database (https://old.tcmsp-e.com/tcmsp.php), selecting compounds with oral bioavailability (OB ≥ 30%) and drug‐likeness (DL ≥ 0.18) to predict their potential targets [19]. The identified targets were mapped to standard gene names using the UniProt database (https://www.uniprot.org), and duplicates were removed to obtain a comprehensive set of CMR active components and their potential targets, which served as candidate genes for further analysis [19].

### 2.13. Collection of PF Disease Targets

The term “pulmonary fibrosis” was used as a keyword to search the GeneCards (https://www.genecards.org/), TTD (https://db.idrblab.net/ttd/), and OMIM (https://www.omim.org/) databases. Data from these sources were exported, duplicates were removed, and the datasets were merged to identify targets associated with PF [20].

### 2.14. Summary of Intersecting Targets and Venn Diagram Creation

The identified PF targets and CMR targets were entered into Venny 2.1.0 (https://bioinfogp.cnb.csic.es/tools/venny) to generate a Venn diagram. The intersection of these targets represents the potential targets of CMR in treating PF.

### 2.15. Construction of the “Drug–Component–Disease Target” Network for CMR

The potential targets of CMR in treating PF were imported into Cytoscape 3.10.1 to construct a drug–component–disease target network. Network analysis modules within Cytoscape were used for visualization to elucidate the relationships between drug components and disease targets.

### 2.16. Construction of Protein–Protein Interaction (PPI) Network

The intersection targets were uploaded to the STRING database (https://string-db.org) with the biological attribute set to “*Homo sapiens*” and a confidence score ≥ 0.4. Unconnected nodes were hidden to construct the PPI network. The PPI data were then imported into Cytoscape 3.10.1 to visualize the network. Core targets within the PPI network were identified using the CytoNCA plugin to explore the potential mechanisms of CMR in treating PF.

### 2.17. Enrichment Analysis

The core targets of CMR in treating PF were imported into the DAVID database (https://david.ncifcrf.gov) for GO and KEGG pathway analysis, with a significance threshold of *p* < 0.05 [21]. The top 10 GO and KEGG pathways were selected and visualized using the Bioinformatics platform (https://www.bioinformatics.com.cn/).

### 2.18. Molecular Docking

Mol2 format files of CMR active components were obtained from the TCMSP database, and PDB format files of the targets were retrieved from the RCSB PDB database (https://www.rcsb.org/). Molecular docking was performed using AutoDockTools 1.5.7, and the results were visualized using PyMOL [22].

### 2.19. Statistical Analysis

All experiments were performed at least in triplicate. Data were analyzed using GraphPad Prism 9.5.0, presented as mean ± SEM. One‐way ANOVA was used for comparisons between multiple groups, with *p* < 0.05 considered statistically significant (^∗^, *p* < 0.05; ^∗∗^, *p* < 0.01; ^∗∗∗^, *p* < 0.001; ^∗∗∗∗^, *p* < 0.0001; and ns, not significant).

## 3. Results

### 3.1. CMR Treatment Attenuates Fibrosis, Inflammation, and Lung Function Loss in the BLM‐Induced Mouse Model

To investigate the therapeutic effects of CMR on PF, we established a PF model in C57BL/6J mice using BLM and sacrificed the mice after 28 days (Figure 1(a)). The experimental groups included a physiological negative control group (NC), a BLM model group, and a CMR treatment group (BLM + CMR). We first assessed functional indicators and pathological changes in the mice. The body weight results showed a significant decline in the BLM group from Day 0 to Day 14, whereas the BLM + CMR group exhibited significant weight gain after Day 7 (Figure 1(b)). Histological analysis revealed no significant pathological changes in the NC group. In contrast, the BLM group showed extensive inflammatory cell infiltration, thickening of alveolar septa, and disordered alveolar structure. Masson’s trichrome staining demonstrated normal lung tissue with minimal blue collagen deposition in the NC group, whereas the BLM group exhibited substantial collagen deposition. The BLM + CMR group significantly reversed these pathological changes, with reduced inflammatory cell infiltration, decreased collagen deposition, and improved alveolar structure (Figures 1(c)–1(d)). Based on H&E and Masson’s trichrome staining, we evaluated the Szapiel score, Ashcroft score, and collagen‐positive area. The BLM group exhibited significantly higher Szapiel scores, Ashcroft scores, and collagen‐positive areas (*p* < 0.05). In contrast, the BLM + CMR group showed significant reductions in these parameters (*p* < 0.05), indicating that CMR effectively mitigated BLM‐induced PF and inhibited pathological lung damage (Figures 1(e)–1(g)). EMT is implicated in the progression of PF. To determine the effects of CMR on EMT in BLM‐induced PF mice, we conducted Western blot analysis on mouse lung tissues. Compared with the NC group, the BLM group exhibited upregulated expression of N‐cadherin and Vimentin and downregulated expression of E‐cadherin. In contrast, CMR treatment downregulated N‐cadherin and Vimentin and upregulated E‐cadherin compared with the BLM group (Figures 1(h)–1(i)). These findings suggest that CMR inhibits the EMT process in BLM‐induced PF mice. To evaluate the impact of CMR on inflammation and fibrosis in a murine model of PF, we conducted RT‐qPCR analyses. The results, presented in Supporting Data (Figures S1A–S1H), demonstrate that CMR significantly inhibits the expression of inflammatory and fibrotic markers in mice with BLM‐induced PF. Additionally, respiratory function tests were performed, with the results shown in Supporting Data (Figures S2A–S2L). These data indicate that CMR effectively mitigates lung functional impairment compared with the BLM treatment group.

FIGURE 1CMR treatment attenuates fibrosis in bleomycin‐induced mouse model. (a) Schematic diagram of the pulmonary fibrosis mouse model and treatment protocol. (b) Line graph showing the trend of body weight changes in mice from Day 0 to Day 28. (c‐d) Histological staining of lung tissues with H&E and Masson’s trichrome. Scale bar: 50 μm. (e) Szapiel score for assessing alveolar inflammation in mice. (f) Ashcroft score for evaluating pulmonary fibrosis, where a score of 0 indicates no fibrosis, and scores from 1 to 8 indicate increasing severity of fibrosis. (g) Analysis of collagen fiber area percentage in Masson’s trichrome staining. (h) Western blot analysis of E‐cadherin, N‐cadherin, and Vimentin protein expression. (i) Quantitative analysis of E‐cadherin, N‐cadherin, and Vimentin protein expression.

**Figure:**
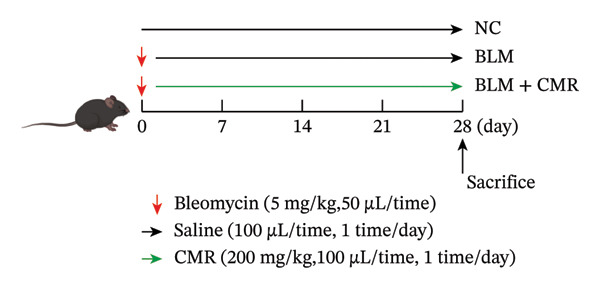
(a)

**Figure:**
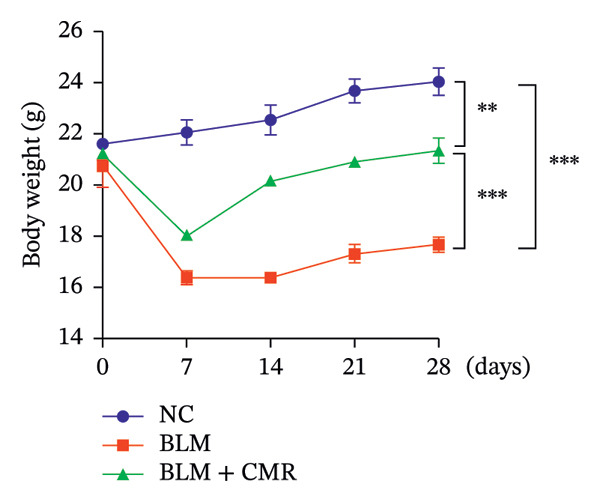
(b)

**Figure:**
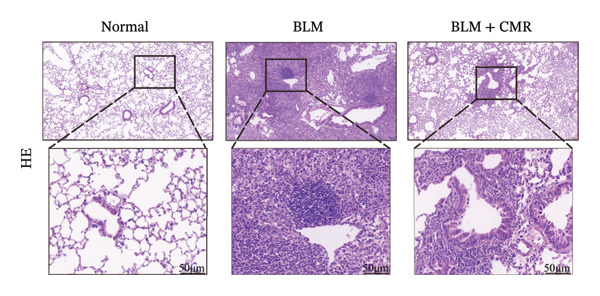
(c)

**Figure:**
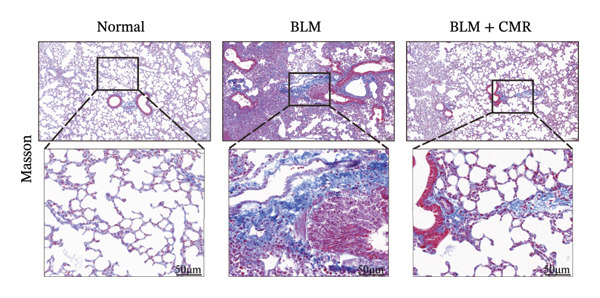
(d)

**Figure:**
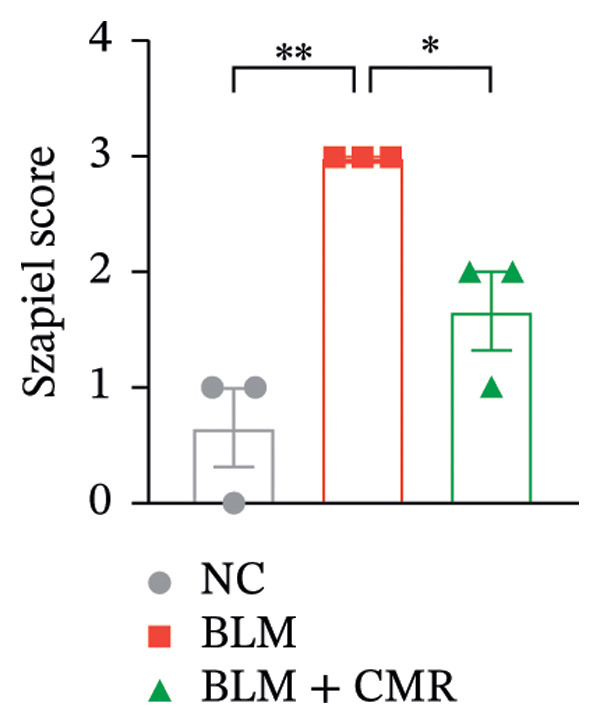
(e)

**Figure:**
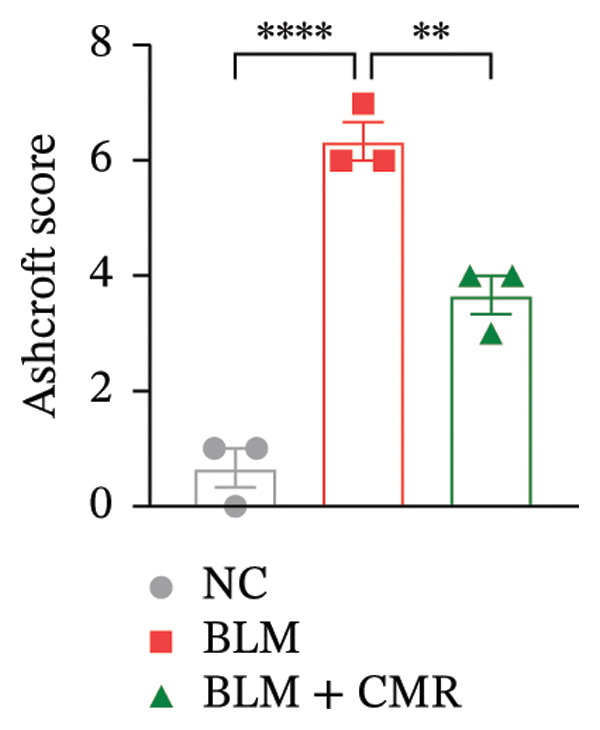
(f)

**Figure:**
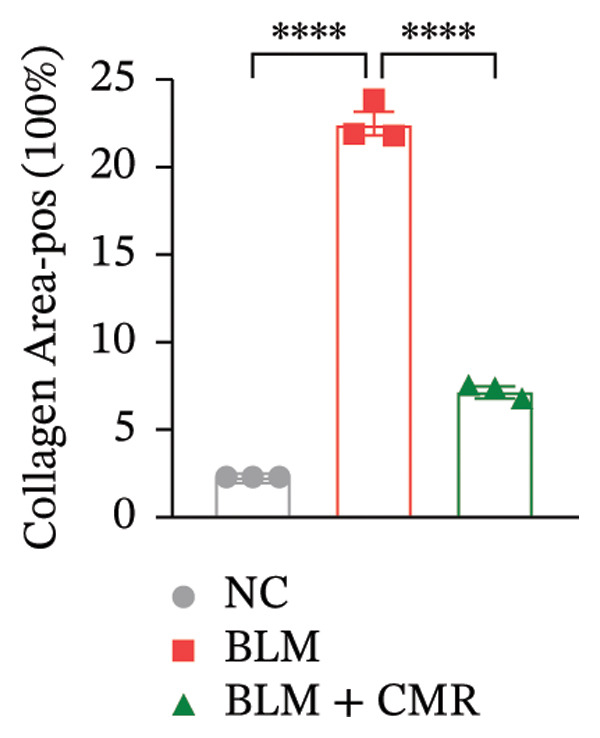
(g)

**Figure:**
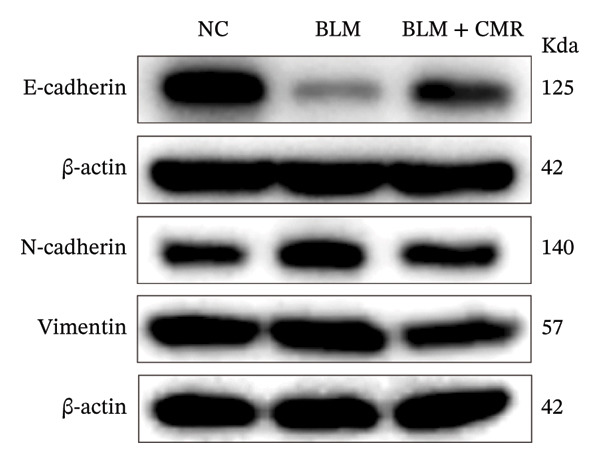
(h)

**Figure:**
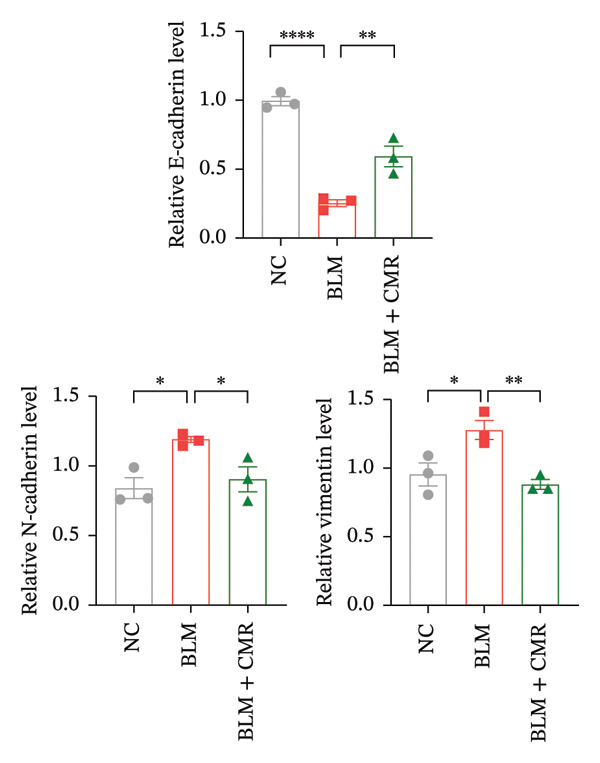
(i)

### 3.2. CMR Inhibits TGF‐β1‐Induced Migration, Proliferation, Inflammation, and Fibrosis in Lung Epithelial Cells

To further elucidate the mechanisms of CMR in PF, we investigated its effects on TGF‐β1‐induced lung epithelial cells. Initial CCK‐8 assays revealed no cytotoxicity at CMR concentrations up to 1000 μg/mL after 24 h. However, prolonged to 48 h of incubation, cell inhibition rates were 25.84% at 250 μg/mL and 45.41% at 1000 μg/mL of CMR (Figure 2(a)). Therefore, we selected 250 μg/mL and 500 μg/mL concentrations for subsequent experiments. CMR significantly attenuated TGF‐β1‐induced migration (Figures 2(b) and 2(c)) and suppressed TGF‐β1‐driven proliferation, as evidenced by reduced colony formation (Figures 2(d) and 2(e)). We further explored CMR’s impact on TGF‐β1‐associated inflammation and fibrosis, which are linked to EMT. CMR treatment for 48 h dose‐dependently reduced TGF‐β1‐induced inflammatory cytokines, including IL‐6, TNF‐α, IL‐1*α*, and IL‐1β (Figures 2(f)–2(i)). Notably, TGF‐β1 upregulated profibrotic proteins N‐cadherin, Vimentin, α‐SMA, Collagen I, and Collagen III, while downregulating E‐cadherin, a hallmark of EMT (Figures 2(j) and 2(k)), Bar graphs further quantified the relative expression levels of E‐cadherin, N‐cadherin, Vimentin, α‐SMA, Collagen I, and Collagen III (Figures S3A–S3F). Collectively, CMR inhibits TGF‐β1‐induced inflammation, fibrosis, migration, and proliferation in lung epithelial cells, highlighting its therapeutic potential in PF.

FIGURE 2CMR inhibits TGF‐β1‐induced migration, proliferation, inflammation, and fibrosis in lung epithelial cells. (a) Cytotoxicity of different doses of CMR on A549 cells after 24 and 48 h of treatment. (b) Effect of CMR on A549 cell migration. Scale bar: 100 μm. (c) Histograms of wound healing rates at 24 and 48 h. (d) Cell colonies on Day 10. (e) Histogram of cell colony numbers on day 10. (f–i) Expression of inflammatory cytokines (IL‐6, TNF‐α, IL‐1*α*, and IL‐1β) after 48 h of CMR treatment. (j)Western blot analysis of E‐cadherin, N‐cadherin, and Vimentin. (k) Western blot analysis of α‐SMA, Collagen I, and Collagen III.

**Figure:**
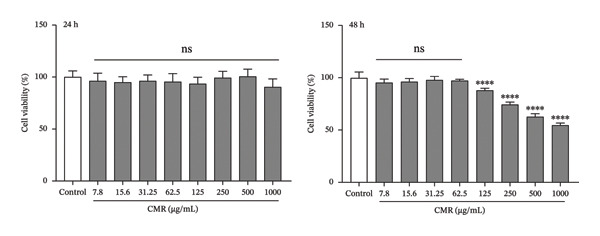
(a)

**Figure:**
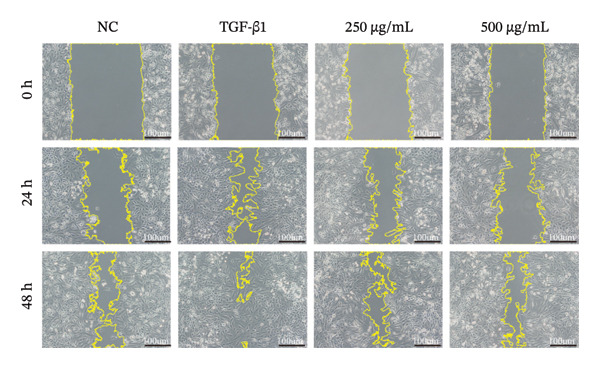
(b)

**Figure:**
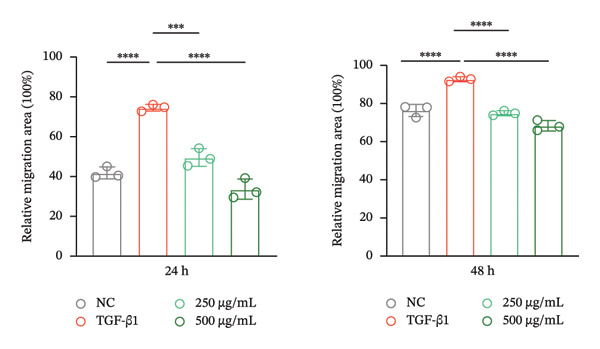
(c)

**Figure:**
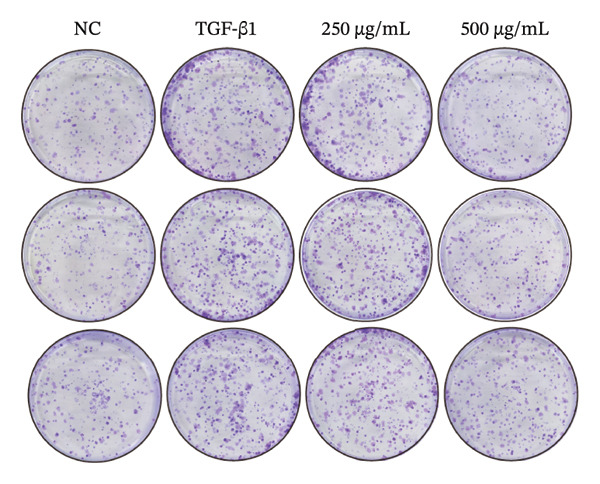
(d)

**Figure:**
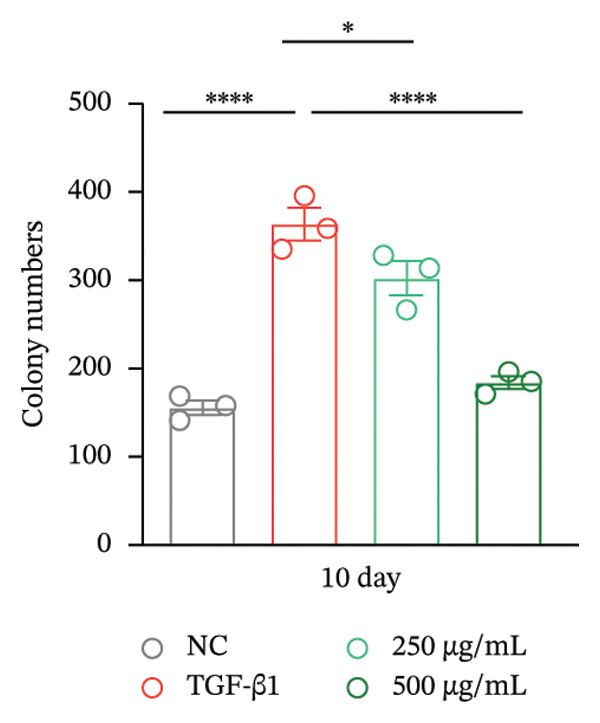
(e)

**Figure:**
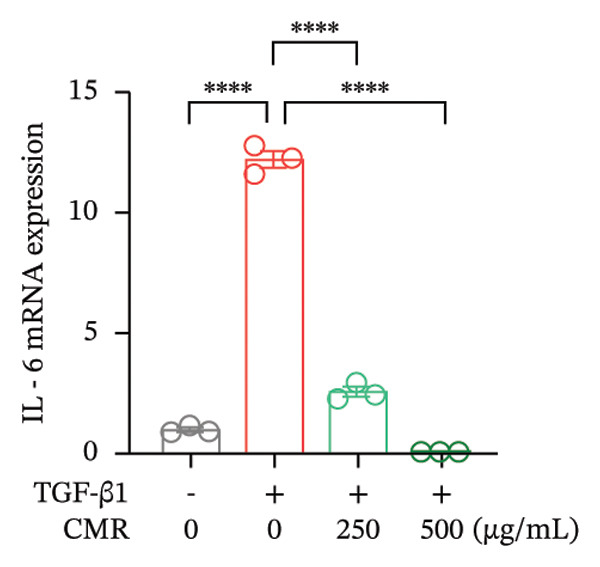
(f)

**Figure:**
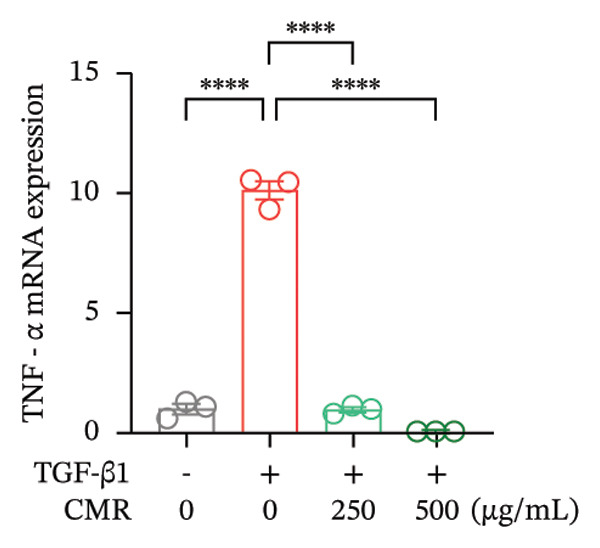
(g)

**Figure:**
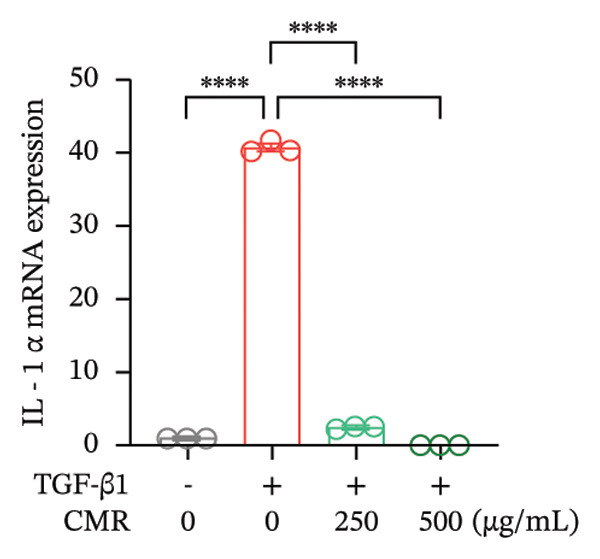
(h)

**Figure:**
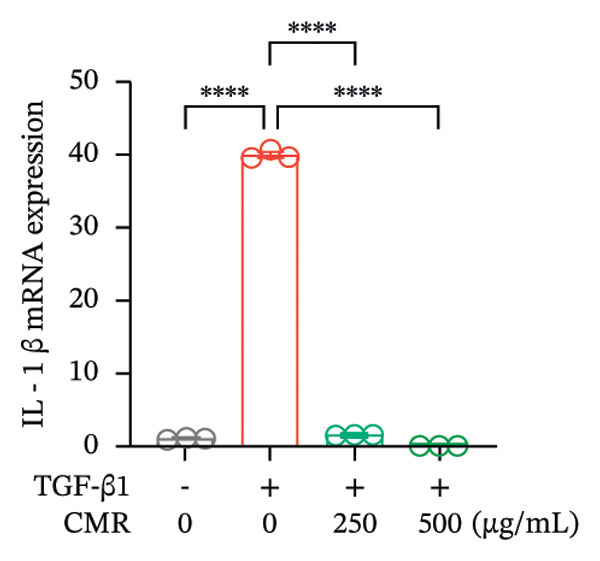
(i)

**Figure:**
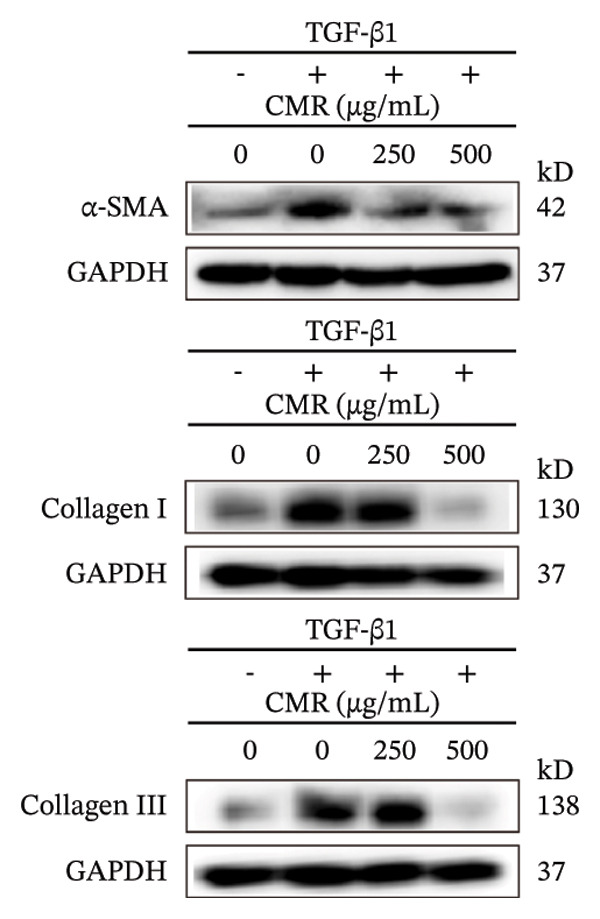
(j)

**Figure:**
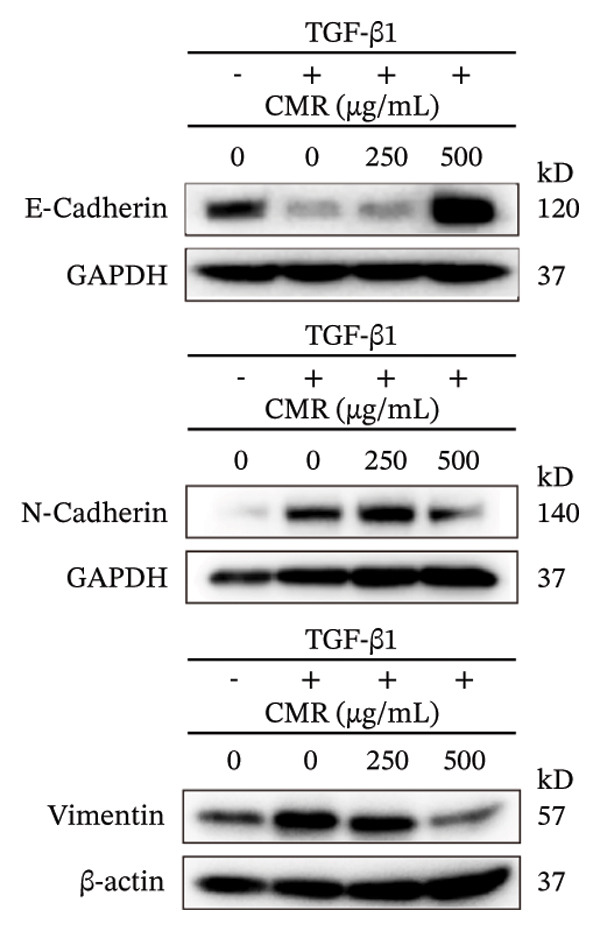
(k)

### 3.3. Network Pharmacology Analysis of the Potential Active Ingredients and Targets of CMR

**FIGURE 3:**
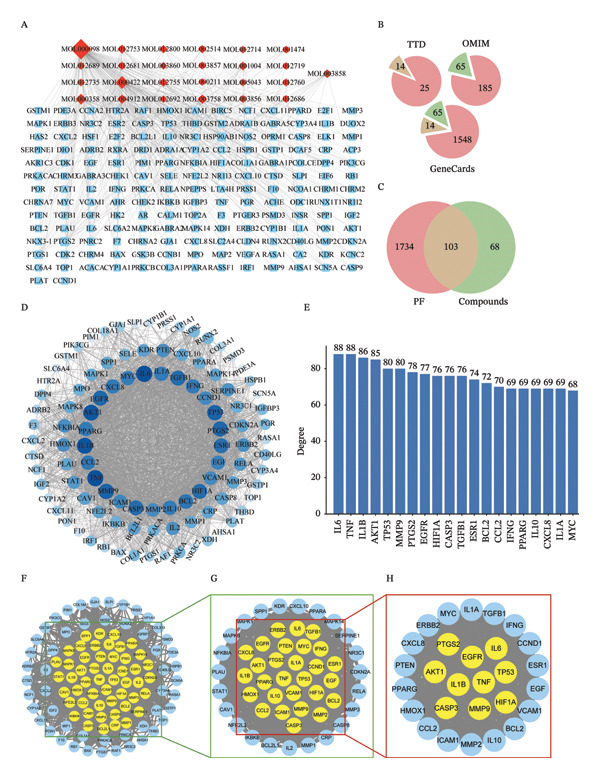
Bioinformatics analysis of Cortex Mori Radicis potential components and targets. (A) Cytoscape‐generated network of CMR compounds and their targets. (B) Targets related to pulmonary fibrosis retrieved from the GeneCards, OMIM, and TTD databases. The diagram illustrates the overlap of targets from each database with those from GeneCards. (C) Venn diagram showing the intersection of CMR drug targets and pulmonary fibrosis targets. (D) Network diagram of the 103 intersection targets. (E) Top 20 targets ranked by degree value. (F–H) Core targets identified using the CytoNCA plugin.

To elucidate the involvement of CMR in the treatment of PF, we employed network pharmacology to identify its potential active components and targets. A total of 25 active components were identified (Supporting Table 2). We obtained 171 unique targets, which were considered candidate genes for further analysis. The top five active components by degree value are quercetin, kaempferol, β‐sitosterol, iristectorigenin (9ci), and glabrone (Figure 3(A)). Using the GeneCards database, we screened for disease‐related targets based on their score values, setting a threshold of > 5 to identify potential targets for PF. This process yielded 1627 disease target genes, with score values ranging from 5.00105381 to 249.2546692. Additionally, we retrieved 39 targets from the TTD database and 250 targets from the OMIM database and obtained a total of 1837 unique PF disease targets (Figure 3(B)). By mapping the 171 CMR‐related targets to the 1837 PF disease targets, we identified 103 common targets (Supporting Table 3). These common targets were visualized using a Venn diagram (Figure 3(C)). To explore the potential mechanisms of CMR in treating PF, the 103 intersection targets of PF and CMR were uploaded to the STRING platform to generate a PPI network, with the protein type set to “*Homo sapiens*.” Core targets were identified based on their degree values, which represent the number of connections each protein has within the network. The network nodes are color‐coded from dark blue (high degree) to light blue (low degree), indicating the relative importance of each protein in the network (Figure 3(D)). The top twenty targets by degree value were ranked (Figure 3(E)). Core targets were further filtered based on degree centrality (DC), betweenness centrality (BC), and closeness centrality (CC), which quantify the importance and influence of nodes within the network. The first filtration resulted in a network with 102 nodes and 2189 edges (Figure 3(F)); the second filtration yielded 51 nodes and 1127 edges (Figure 3(G)); and the third filtration produced 28 nodes and 376 edges (Figure 3(H)). Ultimately, ten high‐performing targets were identified based on degree value and CytoNCA analysis: IL6, TNF, IL1B, AKT1, TP53, MMP‐9, PTGS2, EGFR, HIF1A, and CASP3.

### 3.4. CMR Attenuates PF via Downregulating the PI3K/AKT Pathway

Enrichment analysis was performed on the top 10 entries for visualization. Biological processes were primarily associated with gene expression, inflammatory response, apoptosis regulation, and cell proliferation regulation. Cellular components included extracellular space, cell surface, and secretory granules in the cytoplasm. Molecular functions encompassed enzyme binding, protein binding, and cytokine activity (Figure 4(a)). These results suggest that CMR may exert its anti‐PF effects by modulating cellular inflammatory responses, proliferation, differentiation, and transcriptional regulation. We selected the top 10 signaling pathways related to PF, including cellular senescence, the PI3K‐AKT pathway, the NF‐κB pathway, and apoptosis (Figure 4(b)). To further elucidate the interactions between CMR’s active ingredients and their targets, we constructed a “compound‐target‐pathway” network to illustrate how CMR’s active ingredients interact with key targets and pathways involved in IPF (Figure 4(c)). This network highlights the complex relationships between multiple compounds, their targets, and the signaling pathways they modulate. Additionally, we performed Sankey diagram analysis to quantify and visualize the interactions between CMR’s active ingredients and their targets (Figure 4(d)). This analysis revealed that the most significant interactions were with targets involved in the PI3K/AKT pathway, which further supports the network pharmacology results indicating the central role of this pathway in CMR’s anti‐PF effects. Key targets within these pathways included IL6, IL1B, AKT1, PTGS2, TNF, and EGFR, which align with the results from PPI analysis. Network pharmacology analysis suggests that the PI3K/AKT pathway is a promising molecular mechanism through which CMR exerts its anti‐PF effects. To validate this predicted mechanism, we conducted Western blot analysis in vitro. Consistent with the network pharmacology results, the expression of p‐PI3K and p‐AKT was increased in TGF‐β1‐stimulated A549 cells. Treatment with low (250 μg/mL) and high (500 μg/mL) doses of CMR significantly reduced the expression of these proteins. To confirm that the observed changes in p‐PI3K and p‐AKT represent genuine pathway modulation rather than fluctuations in total protein levels, we repeated the experiments using antibodies against total PI3K and AKT. The results demonstrated that CMR treatment did not significantly alter total PI3K or AKT protein expression (Quantitation Data not shown), while markedly reducing phosphorylated forms (Figures 4(e) and 4(f)).

FIGURE 4CMR attenuates pulmonary fibrosis via downregulating the PI3K/AKT pathway. (a) GO enrichment analysis, including biological processes, cellular components, and molecular functions. (b) Top 10 KEGG enriched pathways. (c) “Compound‐target‐pathway” network in the mechanism of CMR treatment for pulmonary fibrosis. Blue diamond represents CMR compounds, orange rectangles represent targets, and green squares represent enriched pathways. (d) Sankey diagram analysis of drug‐active components and targets of CMR in the treatment of pulmonary fibrosis. (e) Western blot analysis of PI3K, p‐PI3K, AKT and p‐AKT expression. (f) Quantitative analysis of p‐PI3K and p‐AKT protein expression levels.

**Figure:**
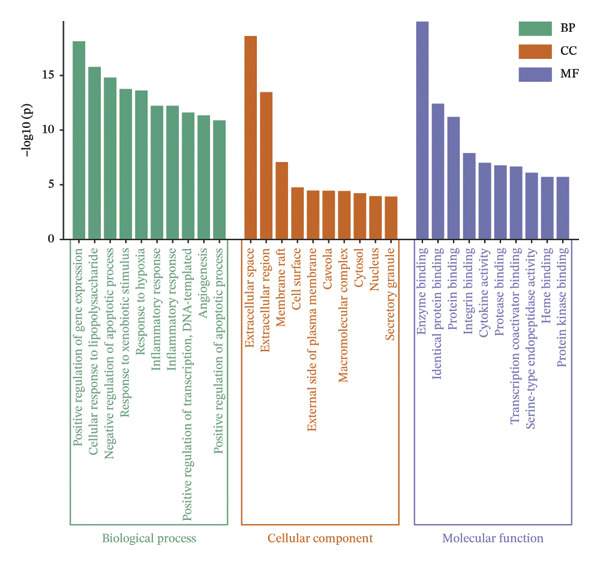
(a)

**Figure:**
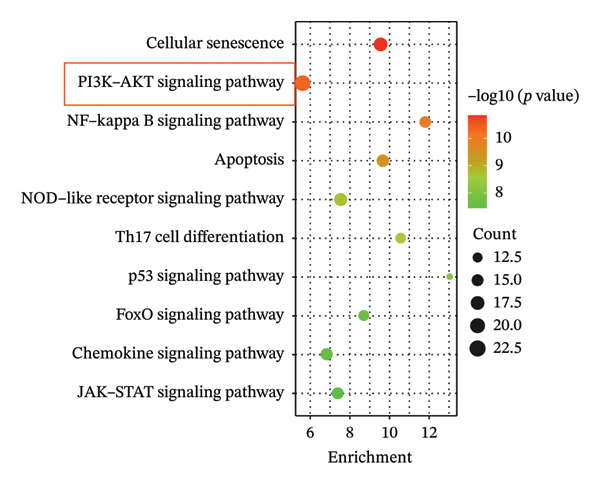
(b)

**Figure:**
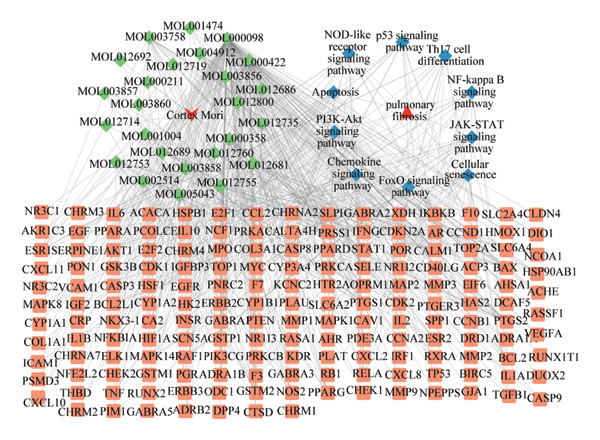
(c)

**Figure:**
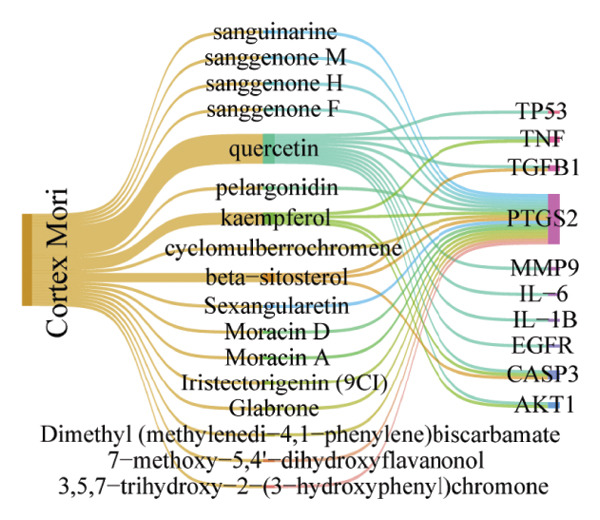
(d)

**Figure:**
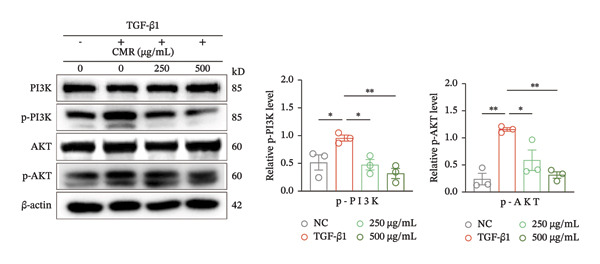
(e)

### 3.5. Molecular Docking of Potential Active Compound in CMR

**FIGURE 5:**
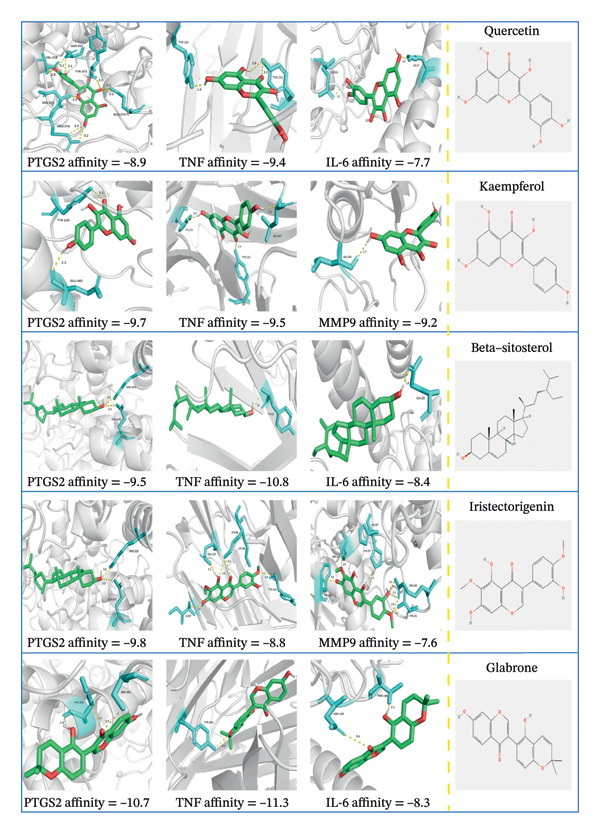
Molecular docking results for the top five core compounds and key targets. The top three targets with the best binding affinity for quercetin were PTGS2, TNF, and IL6; for kaempferol, they were PTGS2, TNF, and MMP‐9; for β‐sitosterol, they were PTGS2, TNF, and IL6; for iristectorigenin, they were PTGS2, TNF, and MMP‐9; and for glabrone, they were PTGS2, TNF, and IL6.

Using network pharmacology, we identified the primary active components of CMR and 10 key targets associated with PF. To evaluate potential therapeutic interactions, molecular docking studies were performed between the top five bioactive compounds, including quercetin, kaempferol, β‐sitosterol, iristectorigenin (9ci), and glabrone, along with the key targets: IL6, TNF, IL1B, AKT1, TP53, MMP‐9, PTGS2, EGFR, HIF1A, and CASP3 for molecular docking studies. A binding energy threshold of ≤ −7.0 kcal·mol^−1^ was applied to assess interactions, which indicate strong affinity. Results revealed that the five compounds exhibited robust binding to four targets: PTGS2, TNF, IL‐6, and MMP‐9. Notably, quercetin, kaempferol, and glabrone showed particularly high affinity across these targets. These findings suggest that CMR’s anti‐PF effects may be mediated through the modulation of PTGS2, TNF, IL‐6, and MMP‐9, which are central to inflammatory and fibrotic pathways (Figure 5).

## 4. Discussion

This study is the first to elucidate the mechanism by which CMR attenuates BLM‐induced PF through the inhibition of the PI3K/AKT signaling pathway. Compared with existing clinical drugs, such as nintedanib and pirfenidone, CMR demonstrates a multitarget regulatory advantage: it significantly reduces the expression of inflammatory cytokines (IL‐6 and TNF‐α) and fibrosis markers (α‐SMA and COL1A1) and blocks the fibrotic process by inhibiting EMT. These findings provide new insights for the development of traditional Chinese medicine formulations with both anti‐inflammatory and antifibrotic functions.

IPF is a disease that progresses from an inflammatory phase to irreversible interstitial fibrosis [23, 24]. Inflammation is the initiating phase of PF, during which persistent alveolar epithelial injury and excessive repair lead to the release of large amounts of inflammatory cytokines, thereby directly or indirectly promoting fibrosis [25]. Studies have shown that inhibiting inflammatory responses can improve tissue pathology and reduce fibrosis [26]. EMT plays a central role in the pathogenesis of PF. In this process, epithelial cells lose their characteristics and polarity and acquire mesenchymal traits with migratory and invasive capabilities. Myofibroblasts derived from EMT are not only key components of fibrotic remodeling but also the main producers of ECM [4, 27]. In the process of PF, fibrosis is usually identified by detecting the loss of epithelial phenotypes (e.g., E‐cadherin) and the increase of mesenchymal phenotypes (e.g., Vimentin and N‐cadherin) [28]. Our study found that CMR reduces alveolar inflammation, inflammatory cell infiltration, and fibrosis and also decreases the expression of IL‐6, IL‐1α, IL‐1β, TNF‐α, and TGF‐β, effectively inhibiting the secretion of inflammatory cytokines, thereby demonstrating that CMR can inhibit inflammatory responses. Also, CMR significantly inhibits the excessive synthesis of ECM and blocks the EMT process.

Through network pharmacology analysis, this study identified 25 active components and 171 potential targets and constructed a drug–component–target network for CMR. Among these, quercetin, kaempferol, β‐sitosterol, iristectorigenin (9ci), and glabrone may play important roles in treating PF. These components exhibit various biological properties, including anti‐inflammatory, antioxidant, and immunomodulatory effects, which may act synergistically to target the pathological processes of PF. For example, quercetin, a natural flavonoid, has been shown to inhibit neuroinflammatory responses and modulate macrophage polarization, thereby improving renal injury and fibrosis [29, 30]. Kaempferol and β‐sitosterol also exhibit similar anti‐inflammatory and antioxidant characteristics and may reduce PF by inhibiting the expression of inflammatory cytokines and oxidative stress responses [31, 32]. Molecular docking results indicate that these five chemical components have good binding activity with PTGS2, TNF, IL‐6, and MMP‐9, providing molecular evidence for their potential role in treating PF. Network pharmacology analysis and experimental validation indicate that the PI3K/AKT signaling pathway is a key molecular mechanism by which CMR exerts its antifibrotic effects. Our study found through Western blot analysis that CMR significantly reduces the expression levels of p‐PI3K and p‐AKT in TGF‐β1‐stimulated A549 cells, thereby inhibiting the activity of this signaling pathway. The PI3K/AKT signaling pathway plays an important role in regulating cell proliferation, survival, and migration, and its abnormal activation is closely related to the occurrence and development of PF [33, 34]. By inhibiting the PI3K/AKT signaling pathway, CMR may alleviate inflammatory responses and the EMT process at multiple levels, thereby mitigating PF.

Although this study has provided a view on the potential mechanisms of CMR in treating PF, there are still several limitations. First, we utilized A549 cells as a surrogate for human alveolar epithelial cells; however, due to their cancer‐derived origin and altered differentiation state, they do not fully recapitulate the characteristics of normal human lung epithelial cells. To address this limitation, future studies will incorporate primary human alveolar epithelial cells or other physiologically relevant epithelial models. Second, although the key pathways were verified in vivo, the downstream effects of pathway inhibition were not fully elucidated. Third, we did not perform an in‐depth analysis of the individual active components of CMR. While we have identified potential drug targets, the specific interactions between individual components and these targets require further investigation. Finally, this study is primarily based on animal models, particularly young mice, whereas IPF occurs predominantly in older adults. Therefore, the therapeutic effectiveness of CMR in human PF still requires confirmation through further clinical trials.

## 5. Conclusion

In summary, our results demonstrated that CMR attenuates PF by inhibiting the PI3K/AKT signaling pathway both in vivo and in vitro experiments, thereby reducing inflammatory responses and blocking EMT process. Through network pharmacology, we conducted a comprehensive study of the compounds and targets of CMR, identifying five primary active components: quercetin, kaempferol, β‐sitosterol, iristectorigenin (9ci), and glabrone and four core targets: PTGS2, TNF, IL‐6, and MMP‐9. This study elucidates the potential therapeutic value of CMR in treating PF and provides a basis for its clinical application.

**Nomenclature**
PF: Pulmonary fibrosis
CMR: Cortex Mori Radicis
EMT: Epithelial–mesenchymal transition
BLM: Bleomycin
PTGS2: Prostaglandin–endoperoxide synthase 2
TNF: Tumor necrosis factor
IL‐6: Interleukin‐6
MMP‐9: Matrix metalloproteinase‐9

## Author Contributions

Tianxiang Qi: writing–review and editing, data curation, and supervision. Bingfeng Ma: writing–original draft and data curation. Yuanyuan Peng: investigation. Yafeng Liu: software. Jianqiang Guo: methodology. Xingyu Yang: investigation. Xuansheng Ding: writing–review and editing. Dingfei Ren: validation. Dong Hu: writing–review and editing.

## Funding

This study was sponsored by the Clinical and Translational Research Project of Anhui Province (202427b10020102, 202427bl0020087, 202427b10020117); Anhui Province Science and Technology Innovation Major Project Plan (202423l10050053); Key Laboratory of Industrial Dust Deep Reduction and Occupational Health and Safety of Anhui Higher Education Institutes (AYZJSGXLK202401002, AYZJSGCLK202402002).

## Ethics Statement

This study was approved by the Medical Ethics Committee of Anhui University of Science and Technology (approval no. HX‐001).

## Conflicts of Interest

The authors declare no conflicts of interest.

## Supporting Information

Additional supporting information can be found online in the Supporting Information section.

## Supporting information


**Supporting Information** Figure S1. CMR inhibits inflammation and fibrosis factor expression in bleomycin‐induced pulmonary fibrosis mice. Figure S2. CMR alleviates lung function loss in bleomycin‐induced pulmonary fibrosis mice. Figure S3. CMR can suppress TGF‐β1‐induced expression of epithelial–mesenchymal transition markers and fibrosis factors in A549 cells. Supporting Table 1. The used primers for RT‐qPCR. Supporting Table 2. The ID, OB and DL of compounds in Cortex Mori Radicis. Supporting Table 3. Potential targets of Cortex Mori Radicis and pulmonary fibrosis.

## Data Availability

The data that support the findings of this study are available from the corresponding author upon reasonable request.
