# Supplementary material for: Cortex Mori Radicis Mitigates Inflammation and Fibrosis in Pulmonary Fibrosis Through PI3K/AKT Pathway Suppression
Source: Can Respir J. 2026 Jun 28;2026:8459298. doi: 10.1155/carj/8459298 (PMC13310380; doi:10.1155/carj/8459298)
Supplement: Supplementary file 1 — Supporting Information Figure S1. CMR inhibits inflammation and fibrosis factor expression in bleomycin‐induced pulmonary fibrosis mice. Figure S2. CMR alleviates lung function loss in bleomycin‐induced pulmonary fibrosis mice. Figure S3. CMR can suppress TGF‐β1‐induced expression of epithelial–mesenchymal transition markers and fibrosis factors in A549 cells. Supporting Table 1. The used primers for RT‐qPCR. Supporting Table 2. The ID, OB and DL of compounds in Cortex Mori Radicis. Supporting Table 3. Potential targets of Cortex Mori Radicis and pulmonary fibrosis. [file CARJ-2026-8459298-s001.zip › Supplementary materials.docx]

**Supplementary Data**


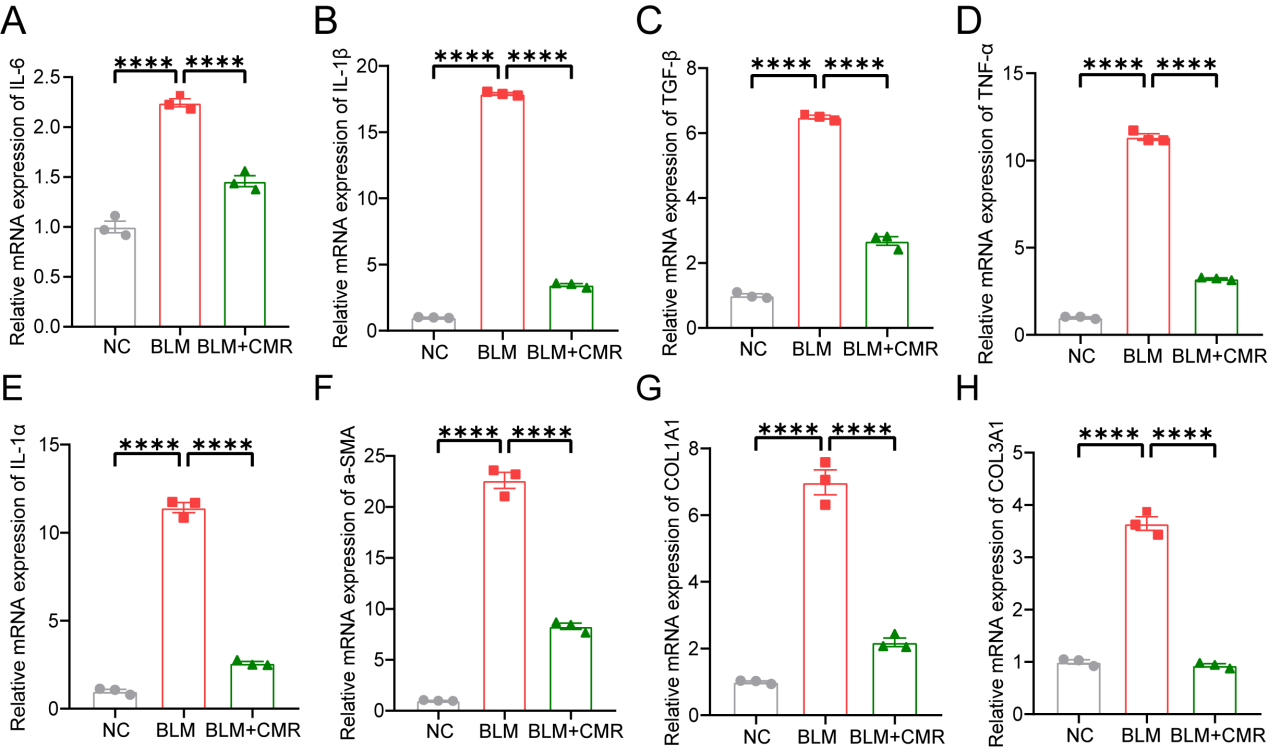


FigureS1. CMR Inhibits Inflammation and Fibrosis Factor Expression in Bleomycin-Induced Pulmonary Fibrosis Mice

(A–E) Levels of IL-6, IL-1β, TGF-β, TNF-α, and IL-1α in mouse lung tissues on day 28.

(F–H) Levels of α-SMA, COL1A1, and COL3A1 in mouse lung tissues on day 28.


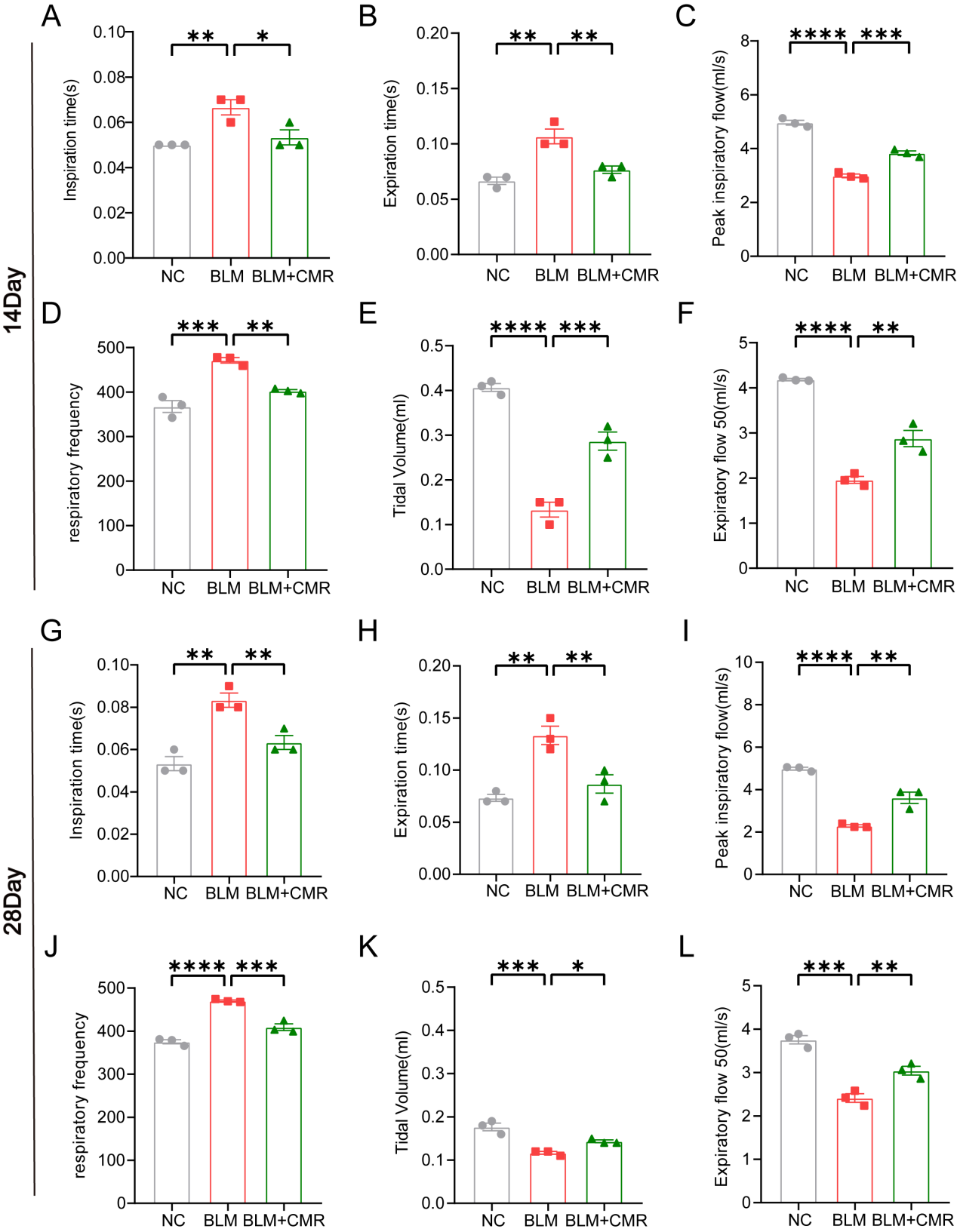


Figure S2. CMR Alleviates Lung Function Loss in Bleomycin-Induced Pulmonary Fibrosis Mice.

(A-F) Respiratory parameters measured on day 14, including expiratory time, inspiratory time, respiratory rate, tidal volume, peak inspiratory flow, and mid-expiratory flow.

(G-L) Respiratory parameters measured on day 28, including expiratory time, inspiratory time, respiratory rate, tidal volume, peak inspiratory flow, and mid-expiratory flow.


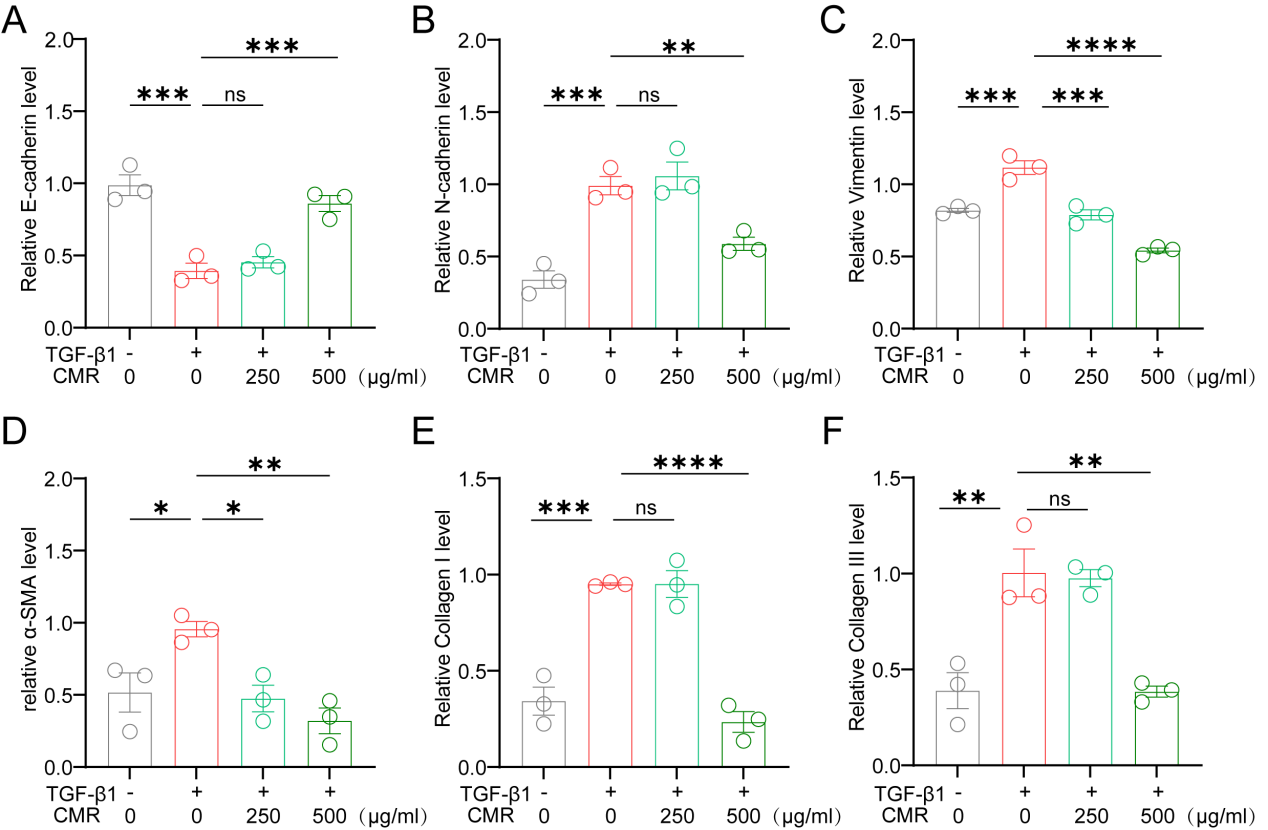


FigureS3. CMR can suppress TGF-β1-induced expression of epithelial-mesenchymal transition markers and fibrosis factors in A549 cells

(A–C) Quantitative analysis of E-cadherin, N-cadherin, and Vimentin protein expression.

(D–F) Quantitative analysis of α-SMA, Collagen I and Collagen III protein expression.

**Supplementary Table 1.**

| **Gene name** | **Forward primer** | **Reverse primer** |
| --- | --- | --- |
| m-GAPDH (123bp) | AGGTCGGTGTGAACGGATTTG | TGTAGACCATGTAGTTGAGGTCA |
| m-IL-1a(126bp) | CGAAGACTACAGTTCTGCCATT | GACGTTTCAGAGGTTCTCAGAG |
| m-IL-1β(89bp） | GCAACTGTTCCTGAACTCAACT | ATCTTTTGGGGTCCGTCAACT |
| m-IL-6(76bp) | TAGTCCTTCCTACCCCAATTTCC | TTGGTCCTTAGCCACTCCTTC |
| m-TNF-α(61bp) | CCCTCACACTCAGATCATCTTCT | GCTACGACGTGGGCTACAG |
| m-TGF-β(133bp) | CTCCCGTGGCTTCTAGTGC | GCCTTAGTTTGGACAGGATCTG |
| m-Col1a1(103bp) | GCTCCTCTTAGGGGCCACT | CCACGTCTCACCATTGGGG |
| m-Col3a1(144bp) | CTGTAACATGGAAACTGGGGAA | CCATAGCTGAACTGAAAACCACC |
| m-a-SMA (102bp) | GTCCCAGACATCAGGGAGTAA | TCGGATACTTCAGCGTCAGGA |

The used primers for RT-qPCR.

| **Supplementary Table 2.**  The ID, OB and DL of compounds in Cortex Mori Radicis. | | | | | |
| --- | --- | --- | --- | --- | --- |
| Mol ID | Molecule Name | MW | AlogP | OB (%) | DL |
| MOL012681 | Dimethyl (methylenedi-4,1-phenylene) biscarbamate | 314.37 | 3.33 | 50.84 | 0.26 |
| MOL012686 | 7-methoxy-5,4'-dihydroxyflavanonol | 302.3 | 2.16 | 51.72 | 0.26 |
| MOL012689 | cyclomulberrochromene | 418.47 | 4.58 | 36.79 | 0.87 |
| MOL012692 | kuwanon D | 422.51 | 4 | 31.09 | 0.8 |
| MOL012714 | Moracin A | 286.3 | 3.38 | 64.39 | 0.23 |
| MOL012719 | moracin O | 326.37 | 3.54 | 62.33 | 0.44 |
| MOL012735 | mulberroside C_qt | 326.37 | 3.54 | 71.39 | 0.46 |
| MOL012753 | sanggenone F | 354.38 | 3.09 | 62.42 | 0.54 |
| MOL012755 | sanggenone H | 354.38 | 3.09 | 37.5 | 0.53 |
| MOL012760 | sanggenone M | 436.49 | 4.4 | 68.29 | 0.85 |
| MOL001474 | sanguinarine | 332.35 | 3.47 | 37.81 | 0.86 |
| MOL000211 | Mairin | 456.78 | 6.52 | 55.38 | 0.78 |
| MOL000358 | beta-sitosterol | 414.79 | 8.08 | 36.91 | 0.75 |
| MOL003758 | Iristectorigenin (9CI) | 330.31 | 2.03 | 71.55 | 0.34 |
| MOL003856 | Moracin B | 286.3 | 3.38 | 55.85 | 0.23 |
| MOL003857 | Moracin C | 310.37 | 5 | 82.13 | 0.29 |
| MOL003858 | Moracin D | 308.35 | 4.2 | 60.93 | 0.38 |
| MOL003860 | Moracin F | 286.3 | 3.38 | 53.81 | 0.23 |
| MOL004912 | Glabrone | 336.36 | 3.12 | 52.51 | 0.5 |
| MOL000098 | quercetin | 302.25 | 1.5 | 46.43 | 0.28 |
| MOL001004 | pelargonidin | 271.26 | 1.93 | 37.99 | 0.21 |
| MOL012800 | 3,5,7-trihydroxy-2-(3-hydroxyphenyl) chromone | 286.25 | 1.77 | 59.71 | 0.24 |
| MOL002514 | Sexangularetin | 316.28 | 1.76 | 62.86 | 0.3 |
| MOL000422 | kaempferol | 286.25 | 1.77 | 41.88 | 0.24 |
| MOL005043 | campest-5-en-3beta-ol | 400.76 | 7.63 | 37.58 | 0.71 |

| **Supplementary Table 3.** Potential targets of Cortex Mori Radicis and pulmonary fibrosis. | | | | | |
| --- | --- | --- | --- | --- | --- |
| NO. | Target | NO. | Target | NO. | Target |
| 1 | PTGS2 | 36 | AKT1 | 70 | GJA1 |
| 2 | PDE3A | 37 | VEGFA | 71 | CYP1A1 |
| 3 | ADRB2 | 38 | CCND1 | 72 | ICAM1 |
| 4 | DPP4 | 39 | BCL2L1 | 73 | IL1B |
| 5 | PTGS1 | 40 | PLAU | 74 | CCL2 |
| 6 | SCN5A | 41 | MMP2 | 75 | SELE |
| 7 | ESR1 | 42 | MMP9 | 76 | VCAM1 |
| 8 | PRSS1 | 43 | MAPK1 | 77 | CXCL8 |
| 9 | NOS2 | 44 | IL10 | 78 | HSPB1 |
| 10 | F10 | 45 | EGF | 79 | IL2 |
| 11 | KDR | 46 | RB1 | 80 | CYP1B1 |
| 12 | PIM1 | 47 | TNF | 81 | PLAT |
| 13 | PIK3CG | 48 | IL6 | 82 | THBD |
| 14 | PRKACA | 49 | CDKN2A | 83 | SERPINE1 |
| 15 | PGR | 50 | AHSA1 | 84 | COL1A1 |
| 16 | CHRM3 | 51 | TP53 | 85 | IFNG |
| 17 | HTR2A | 52 | NFKBIA | 86 | PTEN |
| 18 | SLC6A4 | 53 | XDH | 87 | IL1A |
| 19 | BCL2 | 54 | TOP1 | 88 | MPO |
| 20 | BAX | 55 | RAF1 | 89 | NCF1 |
| 21 | CASP3 | 56 | MMP1 | 90 | GSTP1 |
| 22 | CASP8 | 57 | HIF1A | 91 | NFE2L2 |
| 23 | PRKCA | 58 | STAT1 | 92 | PSMD3 |
| 24 | TGFB1 | 59 | ERBB2 | 93 | COL3A1 |
| 25 | PON1 | 60 | HMOX1 | 94 | CXCL11 |
| 26 | PPARG | 61 | CYP3A4 | 95 | CXCL2 |
| 27 | MAPK14 | 62 | CYP1A2 | 96 | PPARA |
| 28 | MMP3 | 63 | CAV1 | 97 | CRP |
| 29 | RELA | 64 | MYC | 98 | CXCL10 |
| 30 | EGFR | 65 | F3 | 99 | IKBKB |
| 31 | IGFBP3 | 66 | RASA1 | 100 | SPP1 |
| 32 | IGF2 | 67 | GSTM1 | 101 | RUNX2 |
| 33 | CD40LG | 68 | NR3C2 | 102 | CTSD |
| 34 | IRF1 | 69 | NR3C1 | 103 | MAPK8 |
| 35 | SLPI |  |  |  |  |
