# Supplementary material for: Cortex Mori Radicis Mitigates Inflammation and Fibrosis in Pulmonary Fibrosis Through PI3K/AKT Pathway Suppression
Source: Can Respir J. 2026 Jun 28;2026:8459298. doi: 10.1155/carj/8459298 (PMC13310380; doi:10.1155/carj/8459298)
Supplement: Supplementary file 1 — Supporting Information Figure S1. CMR inhibits inflammation and fibrosis factor expression in bleomycin‐induced pulmonary fibrosis mice. Figure S2. CMR alleviates lung function loss in bleomycin‐induced pulmonary fibrosis mice. Figure S3. CMR can suppress TGF‐β1‐induced expression of epithelial–mesenchymal transition markers and fibrosis factors in A549 cells. Supporting Table 1. The used primers for RT‐qPCR. Supporting Table 2. The ID, OB and DL of compounds in Cortex Mori Radicis. Supporting Table 3. Potential targets of Cortex Mori Radicis and pulmonary fibrosis. [file CARJ-2026-8459298-s001.zip › Supplementary Table 2.docx]

| **Supplementary Table 2.** Active Compounds of Cortex Mori Radicis and Their Pharmacological Properties  The ID, OB and DL of compounds in Cortex Mori Radicis. | | | | | |
| --- | --- | --- | --- | --- | --- |
| Mol ID | Molecule Name | MW | AlogP | OB (%) | DL |
| MOL012681 | Dimethyl (methylenedi-4,1-phenylene) biscarbamate | 314.37 | 3.33 | 50.84 | 0.26 |
| MOL012686 | 7-methoxy-5,4'-dihydroxyflavanonol | 302.3 | 2.16 | 51.72 | 0.26 |
| MOL012689 | cyclomulberrochromene | 418.47 | 4.58 | 36.79 | 0.87 |
| MOL012692 | kuwanon D | 422.51 | 4 | 31.09 | 0.8 |
| MOL012714 | Moracin A | 286.3 | 3.38 | 64.39 | 0.23 |
| MOL012719 | moracin O | 326.37 | 3.54 | 62.33 | 0.44 |
| MOL012735 | mulberroside C_qt | 326.37 | 3.54 | 71.39 | 0.46 |
| MOL012753 | sanggenone F | 354.38 | 3.09 | 62.42 | 0.54 |
| MOL012755 | sanggenone H | 354.38 | 3.09 | 37.5 | 0.53 |
| MOL012760 | sanggenone M | 436.49 | 4.4 | 68.29 | 0.85 |
| MOL001474 | sanguinarine | 332.35 | 3.47 | 37.81 | 0.86 |
| MOL000211 | Mairin | 456.78 | 6.52 | 55.38 | 0.78 |
| MOL000358 | beta-sitosterol | 414.79 | 8.08 | 36.91 | 0.75 |
| MOL003758 | Iristectorigenin (9CI) | 330.31 | 2.03 | 71.55 | 0.34 |
| MOL003856 | Moracin B | 286.3 | 3.38 | 55.85 | 0.23 |
| MOL003857 | Moracin C | 310.37 | 5 | 82.13 | 0.29 |
| MOL003858 | Moracin D | 308.35 | 4.2 | 60.93 | 0.38 |
| MOL003860 | Moracin F | 286.3 | 3.38 | 53.81 | 0.23 |
| MOL004912 | Glabrone | 336.36 | 3.12 | 52.51 | 0.5 |
| MOL000098 | quercetin | 302.25 | 1.5 | 46.43 | 0.28 |
| MOL001004 | pelargonidin | 271.26 | 1.93 | 37.99 | 0.21 |
| MOL012800 | 3,5,7-trihydroxy-2-(3-hydroxyphenyl) chromone | 286.25 | 1.77 | 59.71 | 0.24 |
| MOL002514 | Sexangularetin | 316.28 | 1.76 | 62.86 | 0.3 |
| MOL000422 | kaempferol | 286.25 | 1.77 | 41.88 | 0.24 |
| MOL005043 | campest-5-en-3beta-ol | 400.76 | 7.63 | 37.58 | 0.71 |
